# Molecular characterisation of a novel recombinant *Ribgrass mosaic virus* strain FSHS

**DOI:** 10.1186/s12985-016-0487-5

**Published:** 2016-02-18

**Authors:** Ramesh R. Chavan, Michael N. Pearson

**Affiliations:** School of Biological Sciences, The University of Auckland, Private Bag 92019, Auckland, New Zealand

**Keywords:** RMV, RMV cluster, Subgroup 3 tobamovirus, Recombination detection analysis, *Plantago*, *Actinidia*

## Abstract

**Background:**

The genus *Tobamovirus* (*Virgaviridae*) comprises 33 accepted species with the recent addition of eight new viruses and is divided in to three subgroups based on the origin of assembly of the virion and host range. Within the subgroup 1 tobamoviruses the orchid-associated tobamovirus was hypothesized to be a chimeric derivative of recombinations between genome fragments from subgroup 3 and 1. Recombination events involving RdRp, movement and coat protein genes are recorded within subgroup 1 and 2. However natural recombinations have not previously been reported between subgroup 3 tobamoviruses.

**Findings:**

The organization and phylogenetic analyses of the complete genome and the different ORFs placed the new isolate within the *Ribgrass mosaic virus* clade of subgroup 3 tobamoviruses. Recombination detection analyses indicated that the isolate was a chimeric genome with fragments of high similarity to *Ribgrass mosaic virus* (RMV) strains NZ-439 (HQ667978) and Actinidia*-*AC (GQ401365.1) infecting herbaceous *Plantago* sp. and woody *Actinidia* spp., respectively. The recombinant differed across the whole genome by 3-8 % from other published RMV genomes.

**Conclusion:**

In this investigation we report an intra-specific recombination between RMV strains NZ-439 (HQ667978) and Actinidia*-*AC (GQ401365.1), in the replicase component between viral-methyltransferase and viral-helicase regions, resulting in a novel RMV strain FSHS (JQ319720.1) that represents the first described natural recombinant within the RMV cluster of subgroup 3 tobamoviruses.

**Electronic supplementary material:**

The online version of this article (doi:10.1186/s12985-016-0487-5) contains supplementary material, which is available to authorized users.

## Body of text

The viruses of the genus *Tobamovirus* (http://ictvonline.org/virusTaxonomy.asp) that exhibit co-divergence and host-switching have a widespread geographical distribution, an extensive host range and prevail even in abiotic natural ecosystems [8, 15, 21, 24]. Subgroup 1 tobamoviruses mostly infect solanaceous species, with the exception of the orchid infecting *Odontoglossum ringspot virus* (ORSV). The subgroup 2 viruses are highly diverse infecting members of Cactaceae, Cucurbitaceae, Fabaceae, Malvaceae and Passifloraceae, showing host range specificity and serological relatedness that may prelude an increased number of subgroups [14]. Subgroup 3 includes crucifer and plantain infecting viruses. In subgroup 1 the origin-of-assembly (OA) is located in the movement protein (ORF3), while in subgroup 2 it is in the coat protein (ORF4) and in subgroup 3 it’s located in the overlap between ORF3 and ORF4 [8, 15]. The subgroup 3 tobamoviruses are further classified into *Ribgrass mosaic virus* (RMV), *Turnip vein clearing virus* (TVCV) and *Youcai mosaic virus* (YoMV) clusters, based on coat protein phylogeny and host range [12]. Although they were historically considered as Brassicaceae infecting viruses they have a wide host range, with RMV isolates alone infecting taxonomically diverse Rosid and Asterid groups [1], comprising Actinidiaceae, Balsaminaceae, Brassicaceae, Caryophyllaceae, Liliaceae, Plantaginaceae and Scrophulariaceae [4, 12].

RMV was first reported by Holmes from *Plantago lanceolata* L. (Plantaginaceae) and has been described with various acronyms and synonyms over the years [8], with isolates designated as RMV being reported from species of 15 dicot and monocot families [4, 5]. The reference genome of RMV strain: Kons. 1105 isolate R14 (HQ667979) was sequenced from infected *Plantago* sp., followed by isolation of two strains of RMV viz. Kons. 1105-V2 (HQ667980) and NZ-439 (HQ667978) from the same genus [4]. RMV strains Actinidia*-*AC (GQ401365.1) and Actinidia-AD (GQ401366.1) were isolated from infected leaves of *Actinidia chinensis and A. deliciosa* respectively (Actinidiaceae) [4]. These genomes helped to resolve the nomenclature ambiguity and phylogenetic separation of RMV (*sensu stricto*), confirming three distinct clusters within subgroup 3 tobamoviruses [12] and placing several isolates previously regarded as RMV into the YoMV cluster. None of the previously published RMV genomes were identified as recombinants.

In the family *Virgaviridae,* recombinants have been recorded in *Furovirus* [10], *Hordeiviru*s [6], *Tobamovirus* [11, 15] and *Tobravirus* [17] groups. The immediate ancestors of orchid-associated tobamovirus ORSV was hypothesized to be a recombinant between 5′ and 3′ genome fragments from subgroup 3 and 1, respectively [8, 15]. Within subgroup 1 recombination in the replicase read through component in *Tobacco mosaic virus* (TMV) and *Tobacco mild green mosaic virus* (TMGMV) appeared to have resulted in a novel TMGMV isolate H7 [7]. Natural recombinations have also been shown between infectious isolates of TMV and *Tomato mosaic virus* (ToMV), including RdRp, movement and coat protein genes [11]. In subgroup 2 tobamoviruses, different ORFs of the genomes of *Cactus mild mottle virus* (CMMoV) and *Frangipani mosaic virus* (FrMV) were shown to be related to viruses infecting diverse host groups, indicating possible recombinations [16, 20]. However, unlike the diverse subgroups 1 and 2, natural recombinations have not previously been reported between subgroup 3 tobamoviruses.

The RMV isolate FSHS was collected from leaf tissues of *P. major* L., showing mild interveinal mottling, from the Waitakere ranges, Auckland region, New Zealand. The presence of tobamoviruses in symptomatic leaves was confirmed using a TMV immunostrip (Agdia #ISK 37800/0024) and also by indirect ELISA using both TMV and RMV specific antisera [3, 22]. RNA extraction and genome amplification by RT-PCR, cloning, the assembly of consensus sequences and gene translations were conducted as described previously [3, 4]. The genome was amplified as 10 overlapping fragments (using the primer sets listed in Additional file 1: Table S1). Two separate amplicons spanned the individual recombination points and two others amplicons both spanned the whole of the integrated fragment from RMV Actinidia-AC plus adjacent sequence from NZ-439, demonstrating that the complete genome sequence was not an artifact from the assembly of sequence fragments from a mixed infection of two strains.

The complete genome, nucleotide (nt) and amino acid (aa) sequences of all the ORFs and the 5′ and 3′UTR sequences of the new strain (JQ319720.1) were compared with GenBank (http://blast.ncbi.nlm.nih.gov/Blast.cgi), all other complete RMV genome sequences [4] and representative genomes from the TVCV and YoMV clusters [12]. Phylogenetic and molecular evolutionary analyses of the FSHS genome and the complete genomes of 33 representative tobamoviruses, and the relationship of the aa sequences of the four ORFs to selected tobamovirus species were elucidated by the neighbour joining method using MEGA 6.0 [25].

The whole genome and nucleotide sequence of all the ORFs of the strains FSHS (JQ319720.1), Kons. 1105 (HQ667979), NZ-439 (HQ667978) and Actinidia*-*AC (GQ401365.1), representing the RMV cluster, were analysed using seven different recombination detection algorithms viz. RDP, GeneConv, Bootscan, MaxChi, Chimaera, SiScan and 3Seq (http://web.cbio.uct.ac.za/~darren/rdp) for the possible recombination events with RDP4 [19]. Each of the RMV genomes was individually used as the reference sequence for analysis of the potential recombinant genome, the major and minor parents. The recombination breakpoint locations were determined based on highest consensus recombination scores (<0.5) and the least average *P* values obtained for the various algorithms used. Additionally, phylogenetic trees were constructed exclusively for RMV sequences ignoring recombinations, using NJ, UPGMA, FastNJ, ML and Bayesian tree programs built in RDP 4; also ML (GTRCAT) tree incorporating the recombinations [19]. In addition recombination was examined using SplitsTree4 analysis [13].

**Fig. 1:**
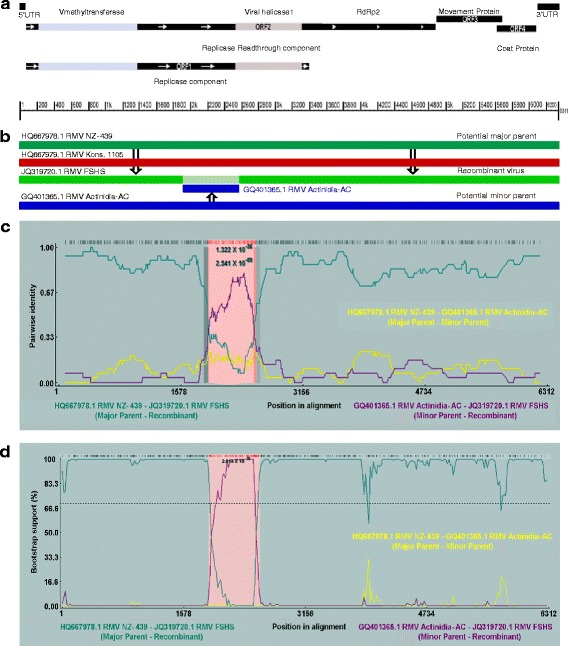
**a** Diagrammatic representation of RMV genome construction; **b** Recombination event in RMV strain FSHS indicating exchange of genome segments; **c** Evidence of recombination from RDP analysis of four RMV genomes; **d** Bootscan evidence for the recombination between RMV genomes (diagrams **b**, **c** and **d** are generated by RDP4). Both the analyses indicate that RMV strain FSHS is a recombinant deriving genome fragments from RMV stains NZ-439 and Actinidia-AC as the major and minor parents. The pink zone in c and d represents the region of recombination between the genomes

The genome of RMV strain FSHS (JQ319720.1), comprising 6311nts, showed the typical organisation of a subgroup 3 tobamovirus with four open reading frames (ORFs), with untranslated regions at 5′ and 3′ ends (Fig. 1a). ORF1 and 2 encode replicase proteins of 125.23 kDa and 181.9 kDa, respectively. ORF1, the putative replicase component, has viral-methyltransferase and viral-helicase domains and codes for 1107 amino acids. ORF2 codes for a replicase read-through component that is associated with RNA-dependent RNA polymerase activity, comprising 1601 amino acids. ORF3 codes for the movement protein, consisting of 267 amino acids (30.1 kDa). ORF4 overlaps ORF3 by 77 nucleotides, spanning nucleotides 5603 to 6076, and codes for 157 amino acids (17.5 kDa) of the coat protein. The 5′ and 3′ UTRs are 67 and 235 nucleotides long, respectively [4].

The complete FSHS genome differs from the reference sequence RMV strain Kons. 1105 isolate R14 (HQ667979) and the Actinidia strains (GQ401365.1 and GQ401366.1) by 8 %, from RMV NZ-439 (HQ667978) by 3 %, and from representative members of TVCV and YoMV clusters by 13 % and 19 %, respectively. The 5′UTR sequence of FSHS was identical to all other sequences in the RMV cluster and differed by 1-9 % from TVCV and YoMV respectively. The 3′UTR differed by <3 % to other sequences in the RMV cluster and 6-8 % from representative members of TVCV and YoMV clusters respectively. Comparisons between the nucleotide (nt) and amino acid (aa) sequences of the four ORFs of strain FSHS (JQ319720.1) and representative species from three clades within subgroup 3 are summarised in Table 1. Sequences of the individual ORFs of FSHS differed by 1-9 % (nt) and 0-3 % (aa) to other sequences within the RMV cluster, the most similar being NZ-439 with 1-3 % (nt) and 0-2 % (aa) difference, and by 12-15 % (nt) and 6-13 % (aa) from TVCV and 17-21 % (nt) and 11-20 % (aa) from YoMV. The new genome exhibited the phylogenetically conserved nucleotide motif identified in the RNA polymerase gene of the tobamovirus [9], between nucleotides 4303-4349 of the replicase read-through component as observed in previously published strains of RMV [4], with an exception at position 4312 where adenine (A) is replaced by thymine (T). In addition the recombinant genome showed two other polymorphisms (position 4332, T instead of A/C/G; position 4345, A instead of G) that are specific to RMV [4].

**Table 1:** Comparison of nucleotide and amino acid sequences of different ORFs of recombinant RMV strain FSHS (accession JQ319720.1) with representative viruses of RMV, TVCV and YOMV clusters of subgroup 3 tobamoviruses

	Virus/sequence id	1	2	3	4	5	6	1	2	3	4	5	6	
		ORF 1						ORF 2						
1	RMV strain FSHS (JQ319720.1)	100	96	91	91	85	79	100	97	91	92	86	80	1
2	RMV strain NZ-439 (HQ667978.1)	**98**	100	89	89	87	79	**99**	100	90	91	87	80	2
3	RMV strain Kons. 1105 (HQ667979.1)	**97**	**96**	100	91	86	79	**98**	**97**	100	92	86	80	3
4	RMV strain Actinidia-AC (GQ401365.1)	**97**	**96**	**98**	100	86	79	**98**	**97**	**98**	100	86	80	4
5	TVCV strain OSU (U03387.1)	**94**	**95**	**94**	**94**	100	80	**94**	**95**	**94**	**94**	100	82	5
6	YoMV (U30944.1)	**88**	**88**	**88**	**88**	**87**	100	**89**	**89**	**90**	**90**	**89**	100	6
		ORF 3						ORF 4						
1	RMV strain FSHS (JQ319720.1)	100	99	93	93	86	79	100	99	96	96	88	83	1
2	RMV strain NZ-439 (HQ667978.1)	**99**	100	94	93	87	79	**100**	100	97	96	89	83	2
3	RMV strain Kons. 1105 (HQ667979.1)	**98**	**99**	100	92	86	79	**99**	**99**	100	96	90	85	3
4	RMV strain Actinidia-AC (GQ401365.1)	**97**	**97**	**97**	100	87	79	**99**	**99**	**99**	100	89	83	4
5	TVCVstrain OSU (U03387.1)	**87**	**88**	**87**	**88**	100	81	**91**	**91**	**92**	**90**	100	86	5
6	YoMV (U30944.1)	**80**	**81**	**80**	**82**	**83**	100	**89**	**89**	**89**	**88**	**88**	100	6
		1	2	3	4	5	6	1	2	3	4	5	6	

*RMV* Ribgrass mosaic virus, *TVCV* Turnip vein-clearing virus, *YoMV* Youcai mosaic virus, Normal and bold numerals indicate nt and aa percentage identities respectively. All the underlined values represent percentage identities of RMV strain FSHS with members of TVCV and YoMV clusters; ORF 1: Replicase component; ORF 2: Replicase read-through component; ORF 3: Movement protein; ORF 4: Coat protein

**Fig. 2:**
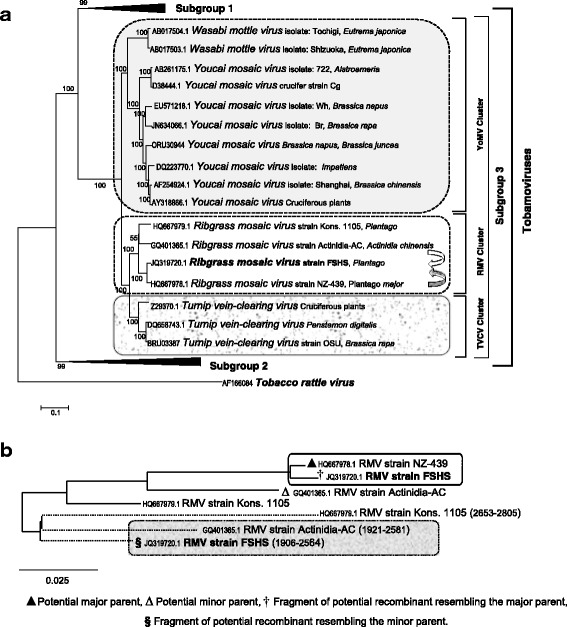
**a** Phylogenetic analysis of complete genome of recombinant RMV strain FSHS with genomes of 33 different tobamoviruses (condensed NJ tree with pairwise gap deletion, substitution including transitions + transversions and uniform rate of evolution options) indicates placement of the new genome in RMV cluster, and also distinct clustering of TVCV, RMV and YoMV genomes within sub group 3. Shaded and open arrows represent the major and minor parents respectively; **b** ML (GTRCAT) tree depicts the phylogenetic relationship of RMV strain FSHS within RMV cluster and the alignment of recombinant fragments with the major and minor parents. Larger fragment of strain FSHS (**†**) aligns with the major parent RMV strain NZ-439 (▲) and 659nts segment of recombinant strain (**§**) aligns with the minor parent RMV strain Actinidia-AC (shaded box)

The phylogenetic analyses of the whole genome of the RMV strain FSHS (JQ319720.1) with 33 different tobamoviruses (Fig. 2a) placed FSHS in the RMV cluster of subgroup 3 tobamoviruses [4, 12], as did the nt and aa sequences of the four individual ORFs (results not shown). Within the RMV cluster strains FSHS and NZ-439 (HQ667978) formed a clade, while strains Kons. 1105 (HQ667979) and Actinidia-AC (GQ401365.1) clustered separately (Fig. 2a). Similar clustering was observed in the phylogenetic trees constructed using exclusively RMV sequences ignoring recombinations, identified by RDP 4 (not shown) [19].

Recombination detection analyses indicated the occurrence of recombination events within the RMV cluster, as summarised in Table 2. The analyses showed that isolate FSHS (JQ319720.1) is a recombinant composed of a 659 nt fragment of the replicase component, between viral-methyltransferase and viral-helicase of the minor parent Actinidia-AC (GQ401365.1) incorporated into the major parent RMV NZ-439 (HQ667978) (Fig. 1b, c, d). The exact position of the beginning and ending break points varied, depending on the reference sequence and algorithm used in the analyses (Table 2). The phylogenetic association of the fragments of the recombinant strain and the major and minor parents are shown in Fig. 2b. The large fragments of strain FSHS (nts 1-1921 and nts 2582-6311) clustered with major parent RMV strain NZ-439 (HQ667978) and the small fragment (nts 1906-2564) with the minor parent RMV Actinidia-AC, at positions between nts 1921-2581 (Fig. 2b). The segments from 5′UTR to the beginning breaking point (1-1905 nt) and the ending break point to 3′UTR (2565-6311 nt) of FSHS showed 99 % (nt) sequence identity with the major parent RMV NZ-439 (HQ667978). The inserted sequence of the recombinant strain revealed 92 % (nt) and 97 % (aa) identity with RMV strain Actinidia-AC (minor parent). SplitsTree4 analysis [13] of the published RMV sequences also confirmed the results of recombination analyses (results not shown).

**Table 2:** Putative recombination events in the genomes and individual open reading frames of viruses of RMV cluster of subgroup 3 tobamoviruses

			Parental isolate		Break points		
	Method	Recombinant	Major	Minor	Beginning	Ending	Average *P*-value
Genome	RDP	RMV FSHS	RMV NZ-439	RMV Actinidia-AC	1906	2564	5×10^-26^
	GeneConv	**”**	**”**	**”**	2148	2479	5×10^-10^
	Bootscan	**”**	**”**	**”**	1905	2564	8×10^-23^ − 5×10^-22^
	MaxChi	**”**	**”**	**”**	1906	2564	3×10^-17^ − 8×10^-14^
	Chimaera	**”**	**”**	**”**	1926	2558	1×10^-06^ − 3×10^-05^
	SciScan	**”**	**”**	**”**	2148	2414	1×10^-10^ − 6×10^-10^
	3Seq	**”**	**”**	**”**	1926	2558	2×10^-21^ − 5×10^-06^
ORF 1-2	RDP	RMV FSHS	RMV NZ-439	RMV Actinidia-AC	1839−1858	2498	5×10^-23^ − 9×10^-20^
	GeneConv	**”**	**”**	**”**	1839	2498	8×10^-11^ − 3×10^-08^
	Bootscan	**”**	**”**	**”**	1839	2498	3×10^-22^ − 2×10^-18^
	MaxChi	**”**	**”**	**”**	1839	2498	6×10^-18^ − 3×10^-12^
	Chimaera	**”**	**”**	**”**	1839	2498	2×10^-16^ − 1×10^-02^
	SciScan	**”**	**”**	**”**	1807−1862	2461−2522	3×10^-11^ − 4×10^-10^
	3Seq	**”**	**”**	**”**	1839	2498	2×10^-22^ − 8×10^-04^
ORF 3		No recombination					
ORF 4		No recombination					

RDP, Bootscan and SiScan are phylogeny-based methods, while GeneCov, MaxChi, and Chimaera are substitution-based methods. 3SEQ recombination detection algorithm tests for mosaicism between three nucleotide sequences. No recombinations were detected using Lard. ‘Major’ and ‘Minor’ parents refer to parental isolates contributing the larger and smaller fractions of recombinant sequence respectively. P-values < 0.05 were considered significant. [Genomes of RMV strains: Kons. 1105-V2 isolate R14 (HQ667980) and Actinidia-AD (GQ401366.1) were not included in the analysis as they were 99 % and 100 % similar to RMV Kons. 1105 isolate R14 (HQ667979) and Actinidia*-*AC (GQ401365.1) respectively]

The fact that RMV is readily mechanically transmissible provides an opportunity for cross-species transmission, possibly resulting in mixed infections and subsequent recombination. Such recombination events might prelude the ability of the new strain to infect both herbaceous and woody asterids expanding the host range, enabling the recombinant to adapt to new micro-replicative niches producing a new range of symptoms, as has been observed in artificially derived tobamovirus recombinants [8, 18, 23, 24]. Recombinations in different ORFs of the genome may variously impact the fitness of recombinants to adapt to environmental change. Capsid genes involved in recombinations are considered to be important as they have multiple roles in the viral life-cycle [2]. Recombinations in the error prone RNA dependent RNA polymerase gene have been reported as a mechanism to restore the levels of precision of viral replication proteins in plant RNA viruses [11] and in tobamoviruses the polymerase domain (54 k) of the gene encoding RdRP has been linked to pathogenicity, based on investigations of artificial chimeras [18]. However the recombination observed in FSHS strain located between viral-methyltransferase and viral-helicase of the replicase component appears to be unique.

## Additional file

Additional file 1: Table S1. Primers sets used to amplify the complete genome of *Ribgrass mosaic virus* strain FSHS isolated from *Plantago* major L. (DOCX 21 kb)

## References

[1] APG 1998 The Angiosperm Phylogeny Group (1998). An ordinal classification for the families of flowering plants. Ann Mo Bot Gard.

[2] Chare ER, Holmes EC (2006). A phylogenetic survey of recombination frequency in plant RNA viruses. Arch Virol.

[3] Chavan RR, Pearson MN, Cohen D (2009). Partial characterization of a novel Tobamovirus infecting *Actinidia chinensis* and *A. deliciosa* (Actinidiaceae) from China. Eur J Plant Pathol.

[4] Chavan RR, Cohen D, Blouin AG, Pearson MN (2012). Characterization of the complete genome of *Ribgrass mosaic virus* isolated from *Plantago major* L. from New Zealand and *Actinidia* spp. from China. Arch Virol.

[5] Cohen D, Chavan RR, Blouin AG, Pearson MN (2012). First report of *Turnip vein clearing virus* and *Ribgrass mosaic virus* from New Zealand. Australasian Plant Dis Notes.

[6] Edwards MC, Petty IT, Jackson AO (1992). RNA recombination in the genome of *Barley stripe mosaic virus*. Virology.

[7] Fraile A, Escriu F, Aranda MA, Malpica JM, Gibbs AJ, Garcı’a-Arenal F (1997). A century of tobamovirus evolution in an Australian population of Nicotiana glauca. J Virol.

[8] Gibbs A (1999). Evolution and origin of tobamoviruses. Phil Trans R Soc Lond B.

[9] Gibbs AJ, Armstrong JS, Gibbs MJ (2004). A type of nucleotide motif that distinguishes tobamovirus species more efficiently than nucleotide signatures. Arch Virol.

[10] Hariri D, Meyer M (2007). New furovirus infecting barley in France closely related to the Japanese soil-borne *Wheat mosaic virus*. Eur J Plant Pathol.

[11] He M, He C-Q, Ding N-Z (2012). Natural recombination between *Tobacco* and *Tomato mosaic viruses*. Virus Res.

[12] Heinze C, Lesemann D-E, Ilmberger N, Willingmann P, Adam G (2006). The phylogenetic structure of the cluster of tobamovirus species serologically related to *Ribgrass mosaic virus* (RMV) and the sequence of *Streptocarpus flower break virus* (SFBV). Arch Virol.

[13] Huson DH, Bryant D (2006). Application of phylogenetic networks in evolutionary studies. Mol Biol Evol.

[14] Kim NR, Hong JS, Song YS, Chung BN, Park JW, Ryu KH (2012). The complete genome sequence of a member of a new species of tobamovirus (*Rattail cactus necrosis-associated virus*) isolated from *Aporcactus flagelliformis*. Arch Virol.

[15] Lartey RT, Voss TC, Melcher U (1996). Tobamovirus evolution: Gene overlaps, recombination, and taxonomic implications. Mol Biol Evol.

[16] Lim MA, Hong JS, Song YS, Ryu KH (2010). The complete genome sequence and genome structure of *Frangipani mosaic virus*. Arch Virol.

[17] MacFarlane SA (1997). Natural recombination among plant virus genomes: evidence from tobraviruses. Semin Virol.

[18] Mansilla C, Sanchez F, Padgett HS, Pogue GP, Ponz F (2009). Chimeras between *Oilseed rape mosaic virus* and *Tobacco mosaic virus* highlight the relevant role of the tobamoviral RdRp as pathogenicity determinant in several hosts. Mol Plant Pathol.

[19] Martin DP, Murrell B, Golden M, Khoosal A, Muhire B (2015). RDP4: Detection and analysis of recombination patterns in virus genomes. Virus Evolution.

[20] Min BE, Song YS, Ryu KH (2009). Complete sequence and genome structure of *Cactus mild mottle virus*. Arch Virol.

[21] Paga’n I, Firth C, Holmes EC (2010). Phylogenetic analysis reveals rapid evolutionary dynamics in the plant RNA virus Genus Tobamovirus. J Mol Evol.

[22] Pearson MN, Chavan RR, Cohen D (2007). Viruses of *Actinidia*: Do They Pose a Threat to Kiwifruit Production?. Acta Hort.

[23] Roossinck MJ (1997). Mechanisms of plant virus evolution. Ann Rev of Phytopathol..

[24] Stobbe AH, Melcher U, Michael W, Palmer MW, Roossinck MJ, Shen G (2012). Co-divergence and host-switching in the evolution of tobamoviruses. Jour Gen Virol.

[25] Tamura K, Stecher G, Peterson D, Filipski A, Kumar S (2013). MEGA6: Molecular Evolutionary Genetics Analysis version 6.0.. Mole Biol Evol.

