# Supplementary material for: Molecular characterisation of a novel recombinant Ribgrass mosaic virus strain FSHS
Source: Virol J. 2016 Feb 18;13:29. doi: 10.1186/s12985-016-0487-5 (PMC4758180; doi:10.1186/s12985-016-0487-5)
Supplement: Additional file 1: Table S1. — Primers sets used to amplify the complete genome of Ribgrass mosaic virus strain FSHS isolated from Plantago major L. (DOCX 21 kb) [file 12985_2016_487_MOESM1_ESM.docx]

**Supplementary Table 1:** Primers sets used to amplify the complete genome of *Ribgrass mosaic virus* strain FSHS isolated from *Plantago* major L.

|  |  |  |  |  |  |
| --- | --- | --- | --- | --- | --- |
| **Primer set** | **Primer** | **Primer name** | **Sequence 5' - 3'** | **Binding Site** | **Product** |
|  |  |  |  |  |  |
| 1 | **+** | RM 1F | GTTTAGTTTTATTGCAACAACA | 1-22 | 305 |
|  | **-** | RM 1R | CAGCGGATTGCGTATGAGTA | 285-305 |  |
|  |  |  |  |  |  |
| 2 | **+** | RM 2F | TACGGAACAGACCCTGATTG | 233-253 | 1204 |
|  | **-** | RM IP R | ACCTAGAATCGCCTTATCTGTG | 1412-1437 |  |
|  |  |  |  |  |  |
| 3 | **+** | RM IP F | TTTTCTTCCATAATGAGAGCAC | 843-863 | 1125 |
|  | **-** | RM 2R | CTCCGCACTTTTTACCTCCT | 1948-1968 |  |
|  |  |  |  |  |  |
| 4 | **+** | RMK F | TTCTCCGATAGATTGGTACTGC | 1644-1666 | 1125 |
|  | **-** | RTO R | TCAACCCTTCGTCAATGAAT | 2749-2769 |  |
|  |  |  |  |  |  |
| 5 | **+** | RM 3F | AACGCATTATCCGAGCTATCA | 1740-1761 | 896 |
|  | **-** | RM 4R | AGCTTCCTTCCCAGGGACTA | 2616-2636 |  |
|  |  |  |  |  |  |
| 6 | **+** | RM 4F | GGTTGTGGAAAGACGAAAGA | 2558-2578 | 1103 |
|  | **-** | RM 5R | TCTTAGGCTTGAGAAAACTTGG | 3639-3661 |  |
|  |  |  |  |  |  |
| 7 | **+** | RM 5F | GCAGTATTCAAGGGAACGAA | 3402-3422 | 906 |
|  | **-** | RM 6R | CTTCAGCCAACCACTCATCT | 4288-4308 |  |
|  |  |  |  |  |  |
| 8 | **+** | RM 7F | AAAGATCAATGCGATTTTCG | 4029-4049 | 712 |
|  | **-** | RM 8R | TCACACAAAGACTCGCGTAA | 4721-4741 |  |
|  |  |  |  |  |  |
| 9 | **+** | RM 8F | TGTGGGGATGACAGTTTAAT | 4464-4484 | 1031 |
|  | **-** | RM 9R | TATCAACCGCTCTAAATGCTTC | 5473-5495 |  |
|  |  |  |  |  |  |
| 10 | **+** | RM 10F | ATCCGTTGCTATGGTTGCTA | 5353-5372 | 958 |
|  | **-** | RM 11R | TGGGCCCCTACCCGGGGTTAGGGA | 6288-6311 |  |
|  |  |  |  |  |  |
|  | | | | | |
| Note: The recombination region recognised encompasses the consensus sequences derived from contiguous segments amplified using primer sets 3, 4, 5 and 6. | | | | | |
